# Supplementary material for: The calcium channel subunit α2δ-3 organizes synapses via an activity-dependent and autocrine BMP signaling pathway
Source: Nat Commun. 2019 Dec 6;10:5575. doi: 10.1038/s41467-019-13165-7 (PMC6898181; doi:10.1038/s41467-019-13165-7)
Supplement: Supplementary file 1 — Supplementary information [file 41467_2019_13165_MOESM1_ESM.pdf]

Supplementary Information

The calcium channel subunit $\alpha_2\delta$-3 organizes synapses via an activity-dependent and autocrine BMP signaling pathway

Hoover et al.

Supplementary Figure 1

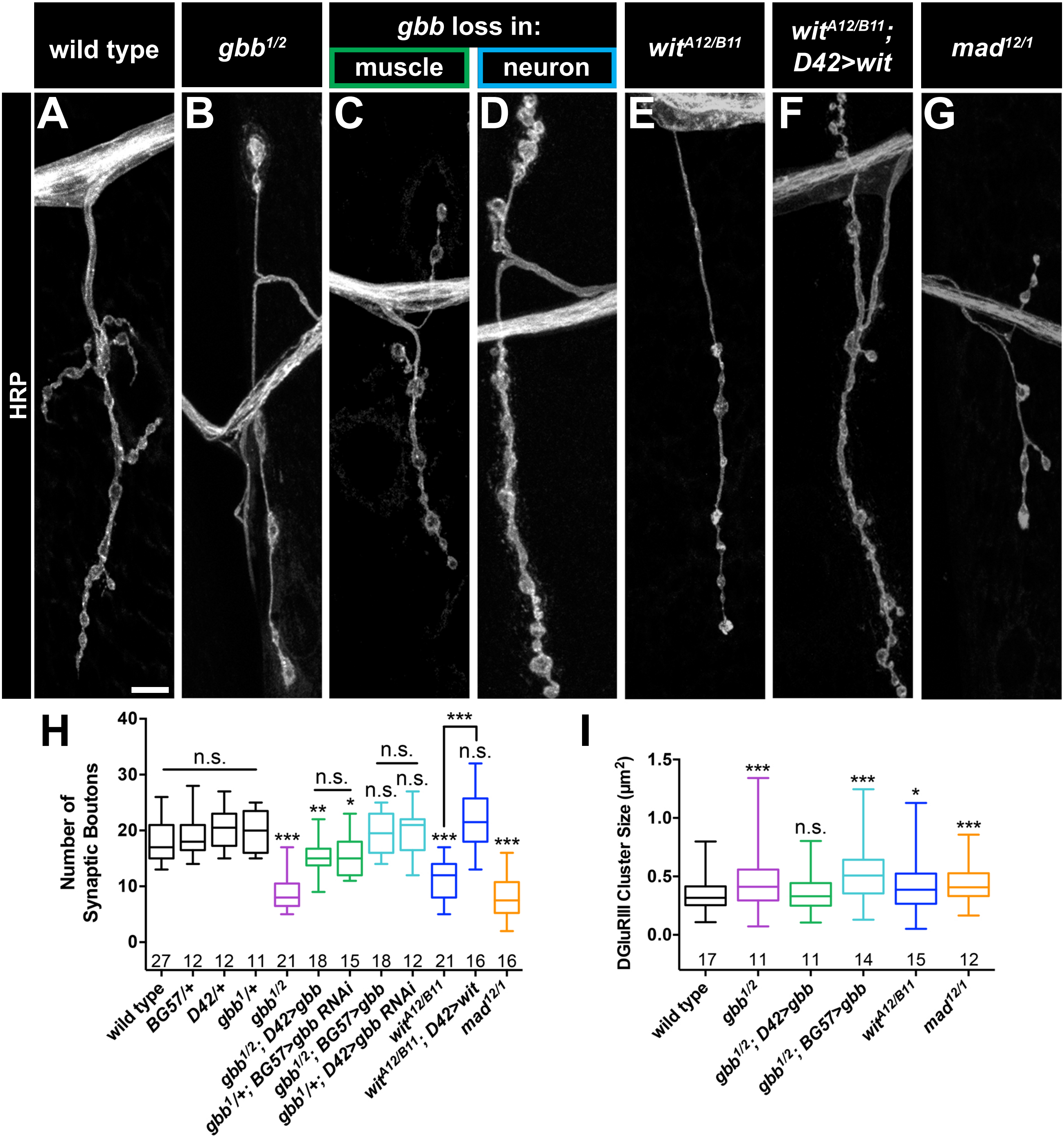

Supplementary Figure 1. Presynaptic Gbb signaling does not regulate NMJ growth. (A-G) Representative z-projections of NMJs of the indicated genotypes labeled with HRP. Scale bar: 5 μm. (H) Quantification of the number of boutons. n is the number of NMJs scored. (I) Quantification of the DGluRIII cluster size in square microns. n is the number of boutons scored. Error bars are min and max data points, and the center line indicates the median. n.s., not significantly different. *, $p<0.05$; **, $p<0.01$; ***, $p<0.001$. All tests are nonparametric Kruskal-Wallis one-way ANOVAs on ranks followed by Dunn's multiple comparison test.

Supplementary Figure 2

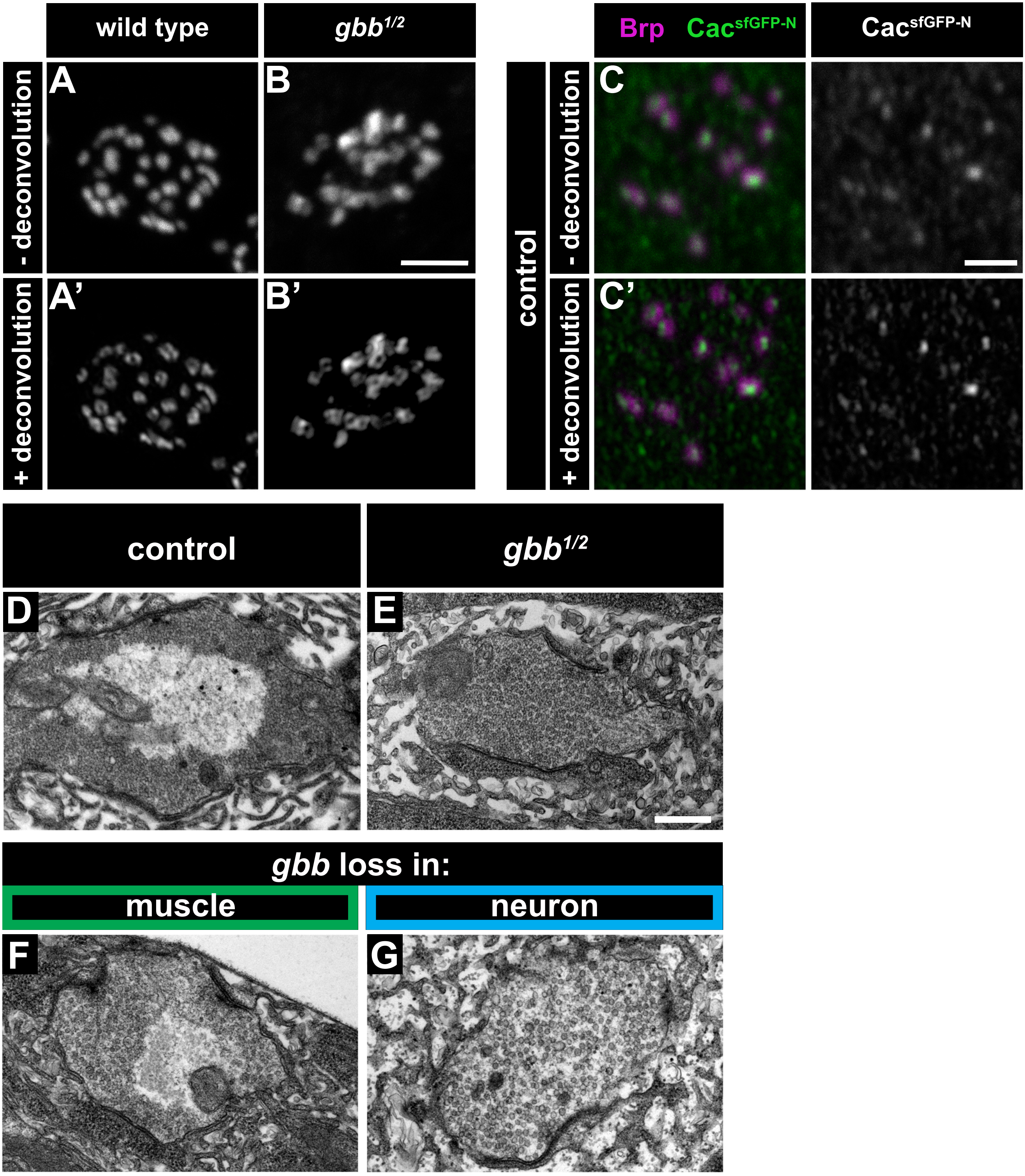

Supplementary Figure 2. Deconvolution allows for visualization of Brp rings, and EM analysis suggests presynaptic Gbb regulates SSV distribution. (A-B′) Representative non-deconvolved and deconvolved z-projections of boutons of indicated genotypes labeled with anti-Brp. Scale bar: 1 μm. (C-C′) Representative non-deconvolved and deconvolved z-projections of boutons of indicated genotypes labeled with Brp (magenta) and endogenously superfolder-GFP-tagged Cacophony (green). Control is $cac^{sfGFP\text{-}N}$. Scale bar: 1 μm. (D-G) Representative transmission electron micrographs of boutons of the indicated genotypes. Scale bar: 1 μm. (D, F) Sections of control and muscle-specific *gbb* LOF boutons reveal synaptic vesicles localized to the bouton perimeter. (E, G) Sections of $gbb^{1/2}$ and neuron-specific *gbb* LOF boutons demonstrate synaptic vesicles filling the entire bouton. *gbb* loss in muscle is $gbb^{1/2}$*; D42>gbb*, and *gbb* loss in neuron is $gbb^{1/2}$*; BG57>gbb*.

Supplementary Figure 3

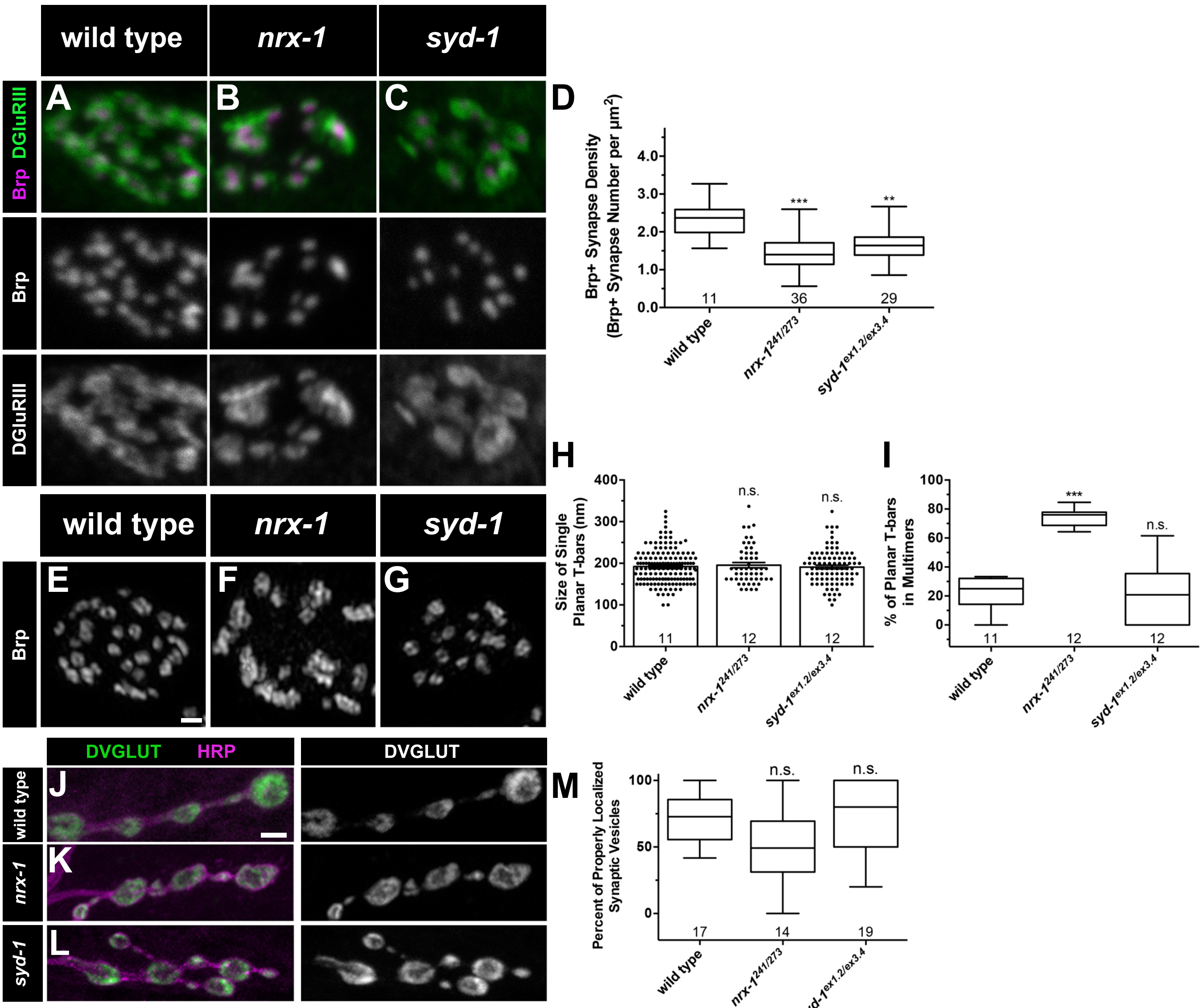

Supplementary Figure 3. Loss of *nrx-1* or *syd-1* results in presynaptic defects. (A-C) Representative z-projections of boutons of the indicated genotypes labeled with Brp (magenta) and DGluRIII (green). Scale bar: 2 μm. (D) Quantification of the Brp+ synapse density. n is the number of boutons scored. (E-G) Representative deconvolved z-projections of boutons of the indicated genotypes labeled with anti-Brp. Scale bar: 1 μm. (H) Quantification of single planar Brp ring diameters. (I) Quantification of the percent of planar Brp rings in multimers in boutons. For Brp ring analyses, n is the number of boutons scored. (J-L) Representative z-projections of boutons of the indicated genotypes labeled with DVGLUT (green) and HRP (magenta). Scale bar: 2 μm. (M) Quantification of the percent of boutons exhibiting properly localized synaptic vesicles. n is the number of NMJs scored. For the bar graph, error bars are mean ± SEM. For all box-and-whisker plots, error bars are min and max data points, and the center line indicates the median. n.s., not significantly different. **, $p<0.01$; ***, $p<0.001$. All tests are nonparametric Kruskal-Wallis one-way ANOVAs on ranks followed by Dunn's multiple comparison test.

Supplementary Figure 4

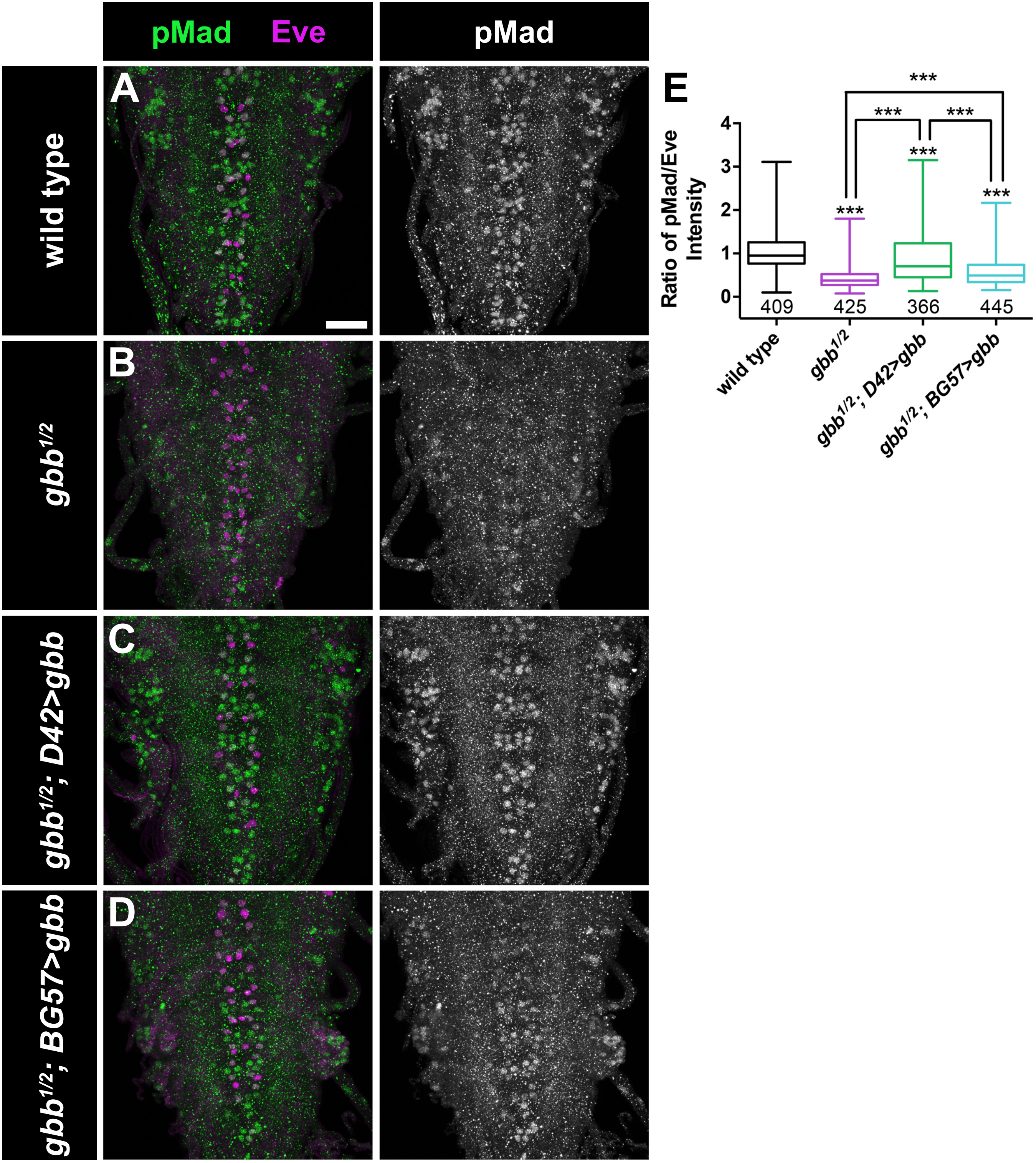

Supplementary Figure 4. Presynaptic Gbb signaling promotes pMad accumulation in the larval VNC. (A-D) Representative z-projections of VNCs labeled with Eve (magenta) and pMad (green) of the indicated genotypes. Scale bar: 25 μm. (E) Quantification of the ratio of pMad to Eve intensity in Eve+ motor neuron cells within the VNC. n is the number of motor neuron cells scored. Error bars are min and max data points, and the center line indicates the median. ***, $p<0.001$. Test is a nonparametric Kruskal-Wallis one-way ANOVA on ranks followed by Dunn's multiple comparison test.

Supplementary Figure 5

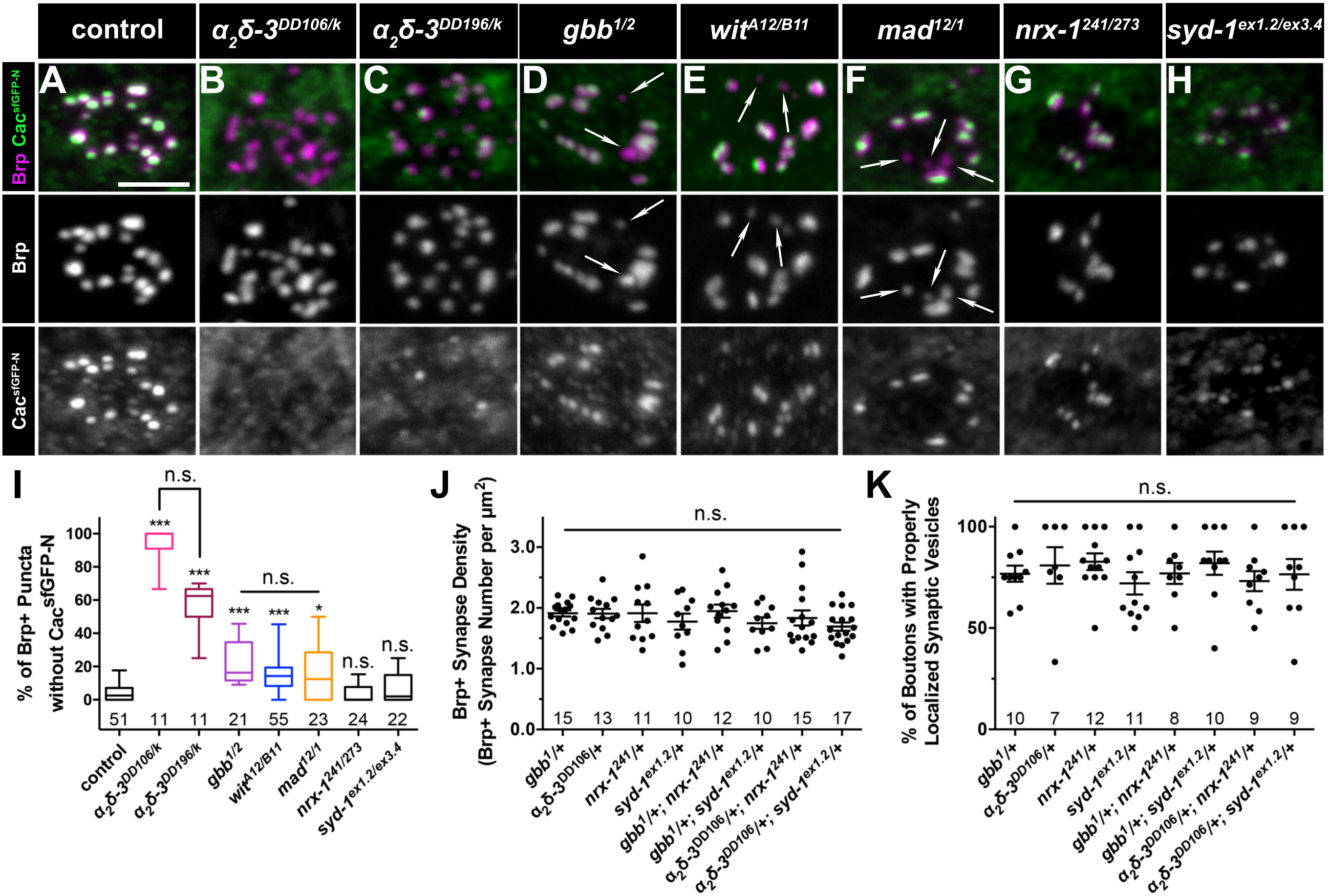

Supplementary Figure 5. $\alpha_2\delta$-3 and BMP pathway members regulate calcium channel localization and do not display genetic interactions with *nrx-1* or *syd-1*. (A-H) Representative z-projections of boutons of the indicated genotypes labeled with Brp (magenta) and endogenously superfolder-GFP-tagged Cacophony (green). Control is $cac^{sfGFP\text{-}N}$. Scale bar: 2 μm. (I) Quantification of the percent of Brp+ puncta without $Cac^{sfGFP\text{-}N}$. n is the number of boutons scored. (J) Quantification of Brp+ synapse density. n is the number of boutons scored. (K) Quantification of the percent of boutons exhibiting properly localized synaptic vesicles. n is the number of NMJs scored. Error bars are min and max data points, and the center line indicates the median. Individual data points are displayed as dots. n.s., not significantly different. *, $p<0.05$; ***, $p<0.001$. All tests are nonparametric Kruskal-Wallis one-way ANOVAs on ranks followed by Dunn's multiple comparison test.

Supplementary Figure 6

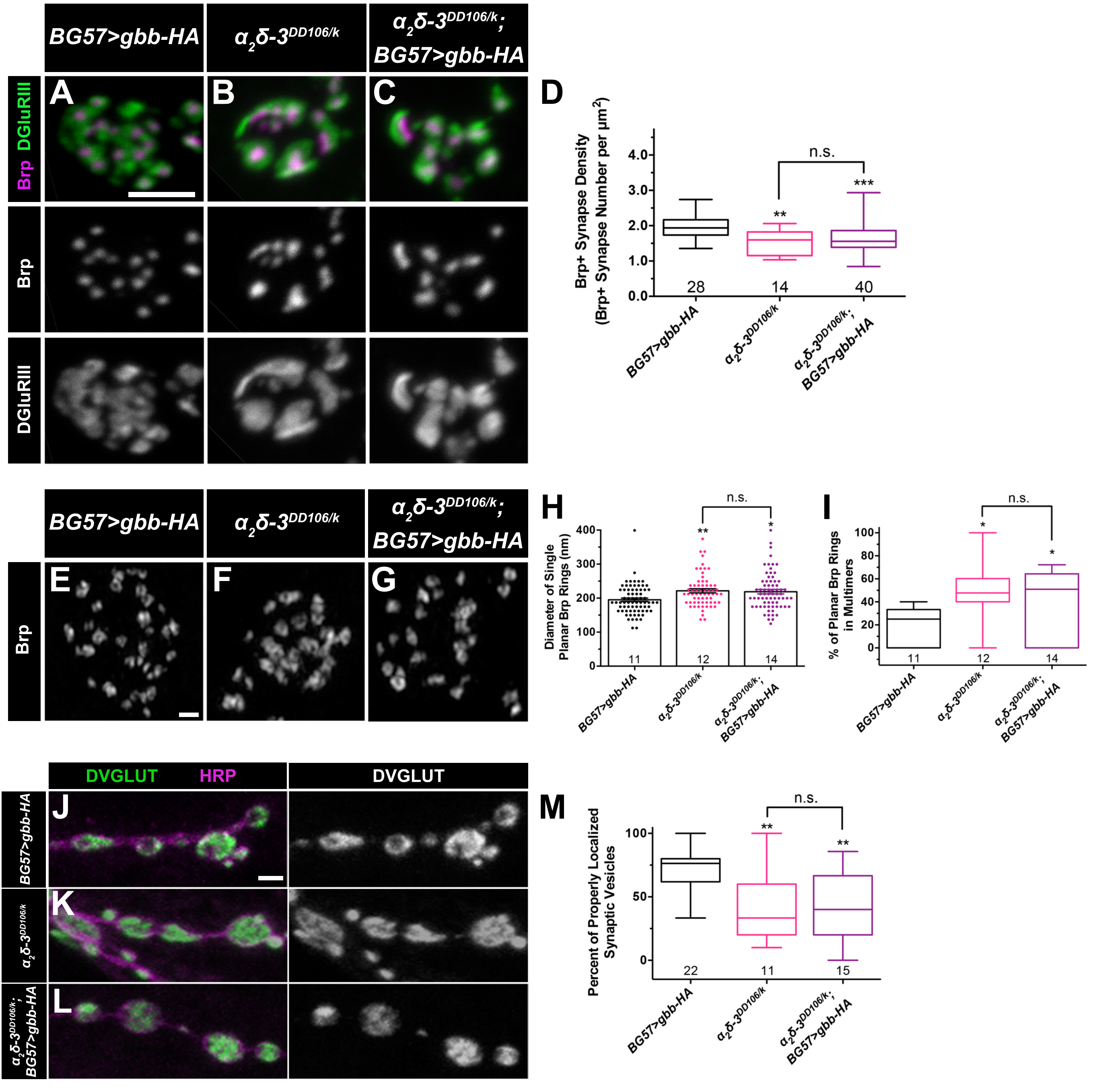

Supplementary Figure 6. Gbb overexpression in muscle does not suppress *$\alpha_2\delta$-3* mutant phenotypes. (A-C) Representative z-projections of boutons of the indicated genotypes labeled with Brp (magenta) and DGluRIII (green). Scale bar: 2 μm. (D) Quantification of the Brp+ synapse density. n is the number of boutons scored. (E-G) Representative deconvolved z-projections of boutons of the indicated genotypes labeled with anti-Brp. Scale bar: 1 μm. (H) Quantification of single planar Brp ring diameters. (I) Quantification of the percent of planar Brp rings in multimers in boutons. For Brp ring analyses, n is the number of boutons scored. (J-L) Representative z-projections of boutons of the indicated genotypes labeled with DVGLUT (green) and HRP (magenta). Scale bar: 2 μm. (M) Quantification of the percent of boutons exhibiting properly localized synaptic vesicles. n is the number of NMJs scored. For the bar graph, error bars are mean ± SEM. For all box-and-whisker plots, error bars are min and max data points, and the center line indicates the median. n.s., not significantly different. *, p<0.05; **, p<0.01; ***, p<0.001. All tests are nonparametric Kruskal-Wallis one-way ANOVAs on ranks followed by Dunn's multiple comparison test.

Supplementary Figure 7

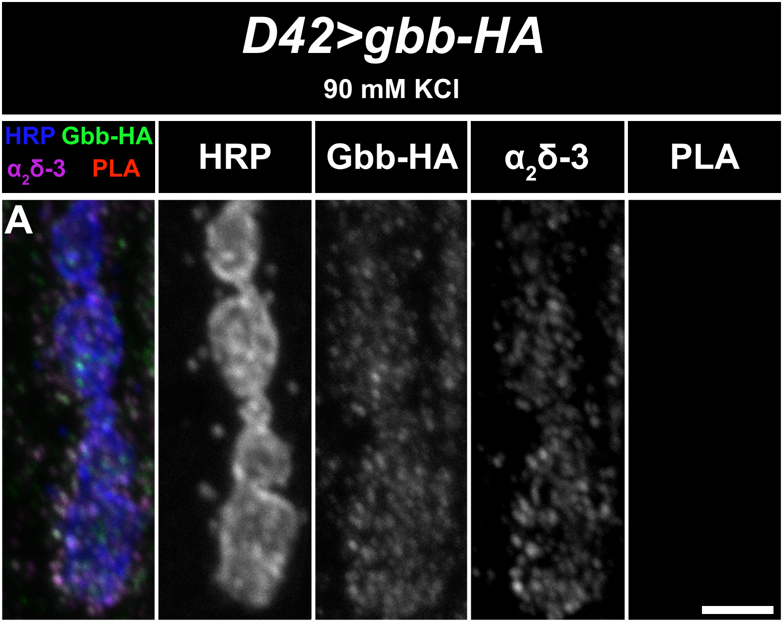

Supplementary Figure 7. No PLA signal is observed in the absence of PLA-specific probes. (A) Representative z-projection of boutons labeled with HRP (blue), Gbb-HA (green), $\alpha_2\delta$-3 (magenta), and PLA-specific probes (red) to detect proximity ligation events. When PLA-specific probes are not added, no PLA signal is observed. Scale bar: 2 μm.

Supplementary Figure 8

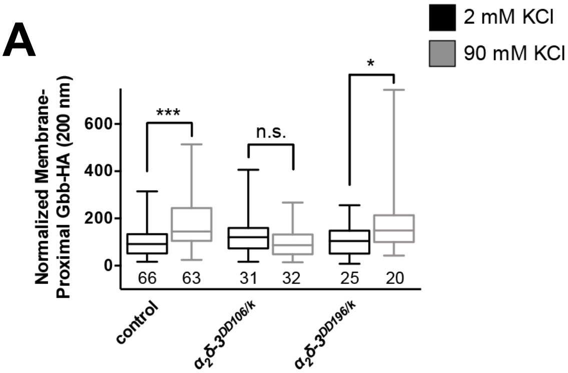

Supplementary Figure 8. $\alpha_2\delta$-3 limits diffusion of Gbb following its activity-dependent release at 200 nm. (A) Quantification of membrane-proximal Gbb-HA (within 200 nm) normalized to control levels before and after neuronal stimulation. n is the number of NMJs scored. Error bars are min and max data points, and the center line indicates the median. n.s., not significantly different. *, $p<0.05$; ***, $p<0.001$ by a two-tailed Mann-Whitney test.
