# Supplementary material for: The calcium channel subunit α2δ-3 organizes synapses via an activity-dependent and autocrine BMP signaling pathway
Source: Nat Commun. 2019 Dec 6;10:5575. doi: 10.1038/s41467-019-13165-7 (PMC6898181; doi:10.1038/s41467-019-13165-7)
Supplement: Supplementary file 3 — Reporting Summary [file 41467_2019_13165_MOESM3_ESM.pdf]

nature research

Corresponding author(s): Heather Broihier
Last updated by author(s): Oct 7, 2019

Reporting Summary

Nature Research wishes to improve the reproducibility of the work that we publish. This form provides structure for consistency and transparency in reporting. For further information on Nature Research policies, see Authors & Referees and the Editorial Policy Checklist.

Statistics

For all statistical analyses, confirm that the following items are present in the figure legend, table legend, main text, or Methods section.

n/a	Confirmed	
☐	☒	The exact sample size (*n*) for each experimental group/condition, given as a discrete number and unit of measurement
☐	☒	A statement on whether measurements were taken from distinct samples or whether the same sample was measured repeatedly
☐	☒	The statistical test(s) used AND whether they are one- or two-sided<br>*Only common tests should be described solely by name; describe more complex techniques in the Methods section.*
☐	☒	A description of all covariates tested
☐	☒	A description of any assumptions or corrections, such as tests of normality and adjustment for multiple comparisons
☐	☒	A full description of the statistical parameters including central tendency (e.g. means) or other basic estimates (e.g. regression coefficient) AND variation (e.g. standard deviation) or associated estimates of uncertainty (e.g. confidence intervals)
☐	☒	For null hypothesis testing, the test statistic (e.g. F, t, r) with confidence intervals, effect sizes, degrees of freedom and P value noted<br>*Give P values as exact values whenever suitable.*
☒	☐	For Bayesian analysis, information on the choice of priors and Markov chain Monte Carlo settings
☒	☐	For hierarchical and complex designs, identification of the appropriate level for tests and full reporting of outcomes
☒	☐	Estimates of effect sizes (e.g. Cohen's *d*, Pearson's *r*), indicating how they were calculated

Our web collection on statistics for biologists contains articles on many of the points above.

Software and code

Policy information about availability of computer code

Data collection	Microscopy images were collected on either the FEI Tecnai G2 Spirit BioTWIN transmission electron microscope or the Zeiss LSM 800 confocal microscope. After imaging samples for Brp rings, Zeiss ZEN deconvolution software was used on images. Locomotion movies were collected on an iPhone 6s. Data collection from images was conducted using ImageJ (version 1.50c4).
Data analysis	All data was statistically analyzed using Prism (version 6.0h) software by GraphPad.

For manuscripts utilizing custom algorithms or software that are central to the research but not yet described in published literature, software must be made available to editors/reviewers. We strongly encourage code deposition in a community repository (e.g. GitHub). See the Nature Research guidelines for submitting code & software for further information.

Data

Policy information about availability of data

All manuscripts must include a data availability statement. This statement should provide the following information, where applicable:

- Accession codes, unique identifiers, or web links for publicly available datasets
- A list of figures that have associated raw data
- A description of any restrictions on data availability

The data that support the findings of this study are available from the corresponding author upon reasonable request.

Field-specific reporting

Please select the one below that is the best fit for your research. If you are not sure, read the appropriate sections before making your selection.

☒ Life sciences ☐ Behavioural & social sciences ☐ Ecological, evolutionary & environmental sciences

For a reference copy of the document with all sections, see nature.com/documents/nr-reporting-summary-flat.pdf

Life sciences study design

All studies must disclose on these points even when the disclosure is negative.

Sample size	No statistical methods were used to determine sample size. Previously published studies using the Drosophila neuromuscular junction provided insights into the current standard of sample sizes needed for experiments. Sample size was determined to be adequate based on the magnitude and consistency of measurable differences between groups.
Data exclusions	For the Brp ring experiments, only planar rings were analyzed, as any rings imaged at a vertical orientation could not be visualized or analyzed. Thus, not all Brp rings within a terminal bouton were analyzed, solely the planar-oriented rings.<br><br>In the pMad experiment, one larval tissue sample was excluded, as the signal to noise ratio was abnormally high. The investigator performing the pMad analysis had never seen such a high signal to noise ratio in any genotype analyzed, which was over hundreds of samples. Thus, this single tissue sample was excluded from the analysis.
Replication	Each experiment presented in the paper was repeated in multiple animals. For electron microscopy and nrx-1/syd-1 genetic interaction experiments, at least two larvae per genotype were analyzed. At least three animals were analyzed for all other experiments. The n for each genotype within an experiment is present in all graphs, and n is defined in the Materials and Methods section or the corresponding figure legend. Replicate experiments were successful and gave similar results. More than one technical replicate was performed for most of the experiments in this study.
Randomization	Drosophila larvae or embryos of the appropriate genotypes were selected at random from a mating cross between male and female adult flies.
Blinding	The investigators were blinded during data collection and analysis for synapse density, Brp ring, and DVGLUT experiments involving wild-type and BMP mutant larvae. Blinding was not performed for additional control larvae in these experiments. Additionally, investigators were blinded for analysis of active zones containing T-bars in electron micrographs, larval locomotion, and Gbb-HA release.

Reporting for specific materials, systems and methods

We require information from authors about some types of materials, experimental systems and methods used in many studies. Here, indicate whether each material, system or method listed is relevant to your study. If you are not sure if a list item applies to your research, read the appropriate section before selecting a response.

Materials & experimental systems

n/a	Involved in the study	
☐	☒	Antibodies
☒	☐	Eukaryotic cell lines
☒	☐	Palaeontology
☐	☒	Animals and other organisms
☒	☐	Human research participants
☒	☐	Clinical data

Methods

n/a	Involved in the study	
☒	☐	ChIP-seq
☒	☐	Flow cytometry
☒	☐	MRI-based neuroimaging

Antibodies

Antibodies used	The following primary antibodies were used: rabbit anti-Bruchpilot (Developmental Studies Hybridoma Bank, NC82) at 1:100, rabbit anti-GluRIII (gift from A. DiAntonio) at 1:2500, rabbit anti-DVGLUT (gift from A. DiAntonio) at 1:10000, rabbit anti-GFP (Abcam, ab6556) at 1:1000, rabbit anti-pMad (Cell Signaling, 41D10) at 1:100, guinea pig anti-Eve (gift from J. Skeath) at 1:2000, rat anti-HA (Roche, 11-867-423-001) at 1:200, guinea pig anti-α2δ-3 (Neely et al., 2010) at 1:500, Alexa Fluor 647-conjugated HRP (Jackson ImmunoResearch) at 1:300, and DyLight-594 anti-HRP (Jackson ImmunoResearch) at 1:300. The following goat species-specific secondary antibodies were used: Alexa Fluor 488 and Alexa Fluor 568 (Invitrogen) at 1:300.
Validation	For each type of antibody used in this study, validation experiments can be found referencing the following citations: anti-Bruchpilot (Wagh et al., 2006), anti-GluRIII (Marrus et al., 2004), anti-DVGLUT (Daniels et al., 2004), anti-GFP to visualize cacsfGFP-N (Gratz et al., 2019), anti-pMad (Smith et al., 2012), anti-Eve (Broihier and Skeath, 2002), anti-HA to visualize Gbb-HA (James et al., 2014), anti-α2δ-3 (Neely et al., 2010), and anti-HRP (Jan and Jan, 1982).

Animals and other organisms

Policy information about studies involving animals; ARRIVE guidelines recommended for reporting animal research

Laboratory animals	This study involved various strains of Drosophila melanogaster. The following stocks were used: OregonR (wild type), BG57Gal4 (Budnik et al., 1996), D42Gal4 (Parkes et al., 1998), Elav-GeneSwitch (Osterwalder et al., 2001), gbb1 and UAS-gbb9.1 (Khalsa et al., 1998), gbb2 (Wharton et al., 1999), UAS-gbb-RNAi (Ballard et al., 2010), witA12, witB11, and UAS-wit (Marqués et al., 2002), mad12 (Sekelsky et al., 1995), mad1 (Takaesu et al., 2005), crimpydel8 (James and Broihier, 2011), cacsfGFP-N (Gratz et al., 2019), α2δ-32 (Ly et al., 2008), α2δ-3DD106, α2δ-3DD196, and α2δ-3k10814 (Dickman et al., 2008), UAS-gbb-1xHA (James et al., 2014), syd-1ex1.2 and syd-1ex3.4 (Owald et al., 2010), nrx273 and nrx241 (Li et al., 2007). Each stock is listed with the proper citation where each stock was first used at the Drosophila neuromuscular junction.<br><br>Both males and females were used in all experiments within this study, except for the electrophysiological experiments where only males were used. Late-stage embryos and wandering third-instar larvae were used in this study.
Wild animals	This study did not involve wild animals.
Field-collected samples	This study did not involve samples from the field.
Ethics oversight	The Institutional Review Board at Case Western Reserve University approved the study protocol.

Note that full information on the approval of the study protocol must also be provided in the manuscript.
